# Therapeutic Potential of Neoechinulins and Their Derivatives: An Overview of the Molecular Mechanisms Behind Pharmacological Activities

**DOI:** 10.3389/fnut.2021.664197

**Published:** 2021-07-16

**Authors:** Javad Sharifi-Rad, Amit Bahukhandi, Praveen Dhyani, Priyanka Sati, Esra Capanoglu, Anca Oana Docea, Ahmed Al-Harrasi, Abhijit Dey, Daniela Calina

**Affiliations:** ^1^Phytochemistry Research Center, Shahid Beheshti University of Medical Sciences, Tehran, Iran; ^2^G.B Pant National Institute of Himalayan Environment, Almora, India; ^3^Institute of Himalayan Bioresource Technology, Palampur, India; ^4^Department of Biotechnology Graphic Era University, Dehradun, India; ^5^Food Engineering Department, Faculty of Chemical and Metallurgical Engineering, Istanbul Technical University, Maslak, Turkey; ^6^Department of Toxicology, University of Medicine and Pharmacy of Craiova, Craiova, Romania; ^7^Natural and Medical Sciences Research Centre, University of Nizwa, Nizwa, Oman; ^8^Department of Life Sciences, Presidency University, Kolkata, India; ^9^Department of Clinical Pharmacy, University of Medicine and Pharmacy of Craiova, Craiova, Romania

**Keywords:** neoechinulins, alkaloid, fungus, *in vitro*, *in vivo*, anticancer, antiviral, anti-inflammatory

## Abstract

Neoechinulins are diketopiperazine type indole alkaloids that demonstrate radical scavenging, anti-inflammatory, antiviral, anti-neurodegenerative, neurotrophic factor-like, anticancer, pro-apoptotic, and anti-apoptotic properties. An array of neoechinulins such as neoechinulins A-E, isoechinulins A-C, cryptoechunilin have been isolated from various fungal sources like *Aspergillus* sp., *Xylaria euglossa, Eurotium cristatum, Microsporum* sp., etc. Besides, neoechinulin derivatives or stereoisomers were also obtained from diverse non-fungal sources *viz. Tinospora sagittata, Opuntia dillenii, Cyrtomium fortunei, Cannabis sativa*, and so on. The main purpose of this review is to provide update information on neoechinulins and their analogues about the molecular mechanisms of the pharmacological action and possible future research. The recent data from this review can be used to create a basis for the discovery of new neoechinulin-based drugs and their analogues in the near future. The online databases PubMed, Science and Google scholar were researched for the selection and collection of data from the available literature on neoechinulins, their natural sources and their pharmacological properties. The published books on this topic were also analysed. *In vitro* and *in vivo* assays have established the potential of neoechinulin A as a promising anticancer and anti-neuroinflammatory lead molecule. Neoechinulin B was also identified as a potential antiviral drug against hepatitis C virus. Toxicological and clinical trials are needed in the future to improve the phyto-pharmacological profile of neoquinolines. From the analysis of the literature, we found that neoechinulins and their derivatives have special biological potential. Although some modern pharmacological analyzes have highlighted the molecular mechanisms of action and some signalling pathways, the correlation between these phytoconstituents and pharmacological activities must be validated in the future by preclinical toxicological and clinical studies.

## Introduction

Neoechinulins are alkaloids and are consisted of three structural moieties *viz*. an indole, an isoprenyl and a diketopiperazine moiety. There are several neoechinulins and related metabolites including neoechinulins A-E, isoechinulins A-C, cryptoechunilin etc. (1). Neoechinulins have been isolated from *Aspergillus* sp. (2–5), ascomycete *Xylaria euglossa* (6), and some others (7, 8).

Echinulin, preechinulin, and neoechinulin E were also obtained as minor metabolites in the extracts of *Eurotium amstelodami* and *E. rubrum* cultures (9). Although rarely observed, neoechinulins and their derivatives or stereoisomers were also isolated from different sources. Stereoisomers of diketopiperazine indole alkaloids namely (12S, 22R)-Dihydroxyisoechinulin A, (12S, 22S)-Dihydroxyisoechinulin A, (12R)-Neoechinulin A, and (12S)-Neoechinulin A were isolated from hemp seed (10). Their presence in different plant sources including Tinosporae Radix (the roots of *Tinospora sagittata*) (11), *Opuntia dillenii* (12), *Cyrtomium fortunei* (13) etc. have also been reported. Table 1 presents the plant species as firstly reported natural sources of neoechinulins.

**Table 1:** The most important natural sources of neoechinulins.

**Species**	**Family**	**Plant part**	**Extraction**	**Isolated compounds**	**References**
*Aconitum carmichaelii* Debeaux	Ranunculaceae	Root	Ethanolic extract	Neoechinulin A 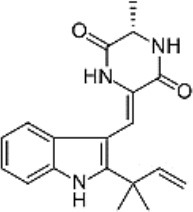	(14)
*Portulaca oleracea* L.	Portulacaceae	Whole	Aqueous extract	Oleraindole A 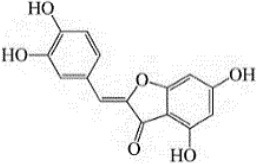 Neoechinulin A 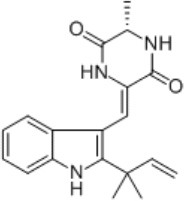 Neoechinulin D 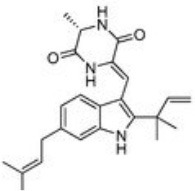 Isoechinulin A 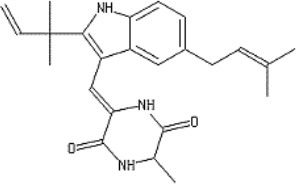 Echinulin 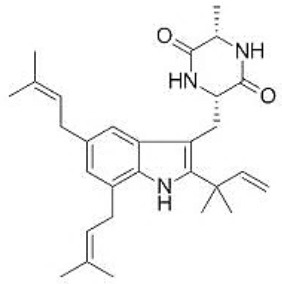	(15)
Marine fungi	*Eurotium* sp. SF-5989	Sponges		Neoechinulin A 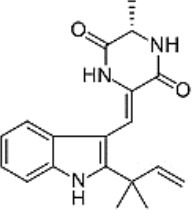 Neoechinulin B 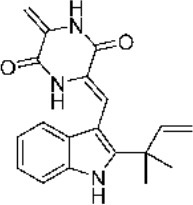	(16)
Marine fungi	*Eurotium. Chevalieri* *Eurotium rubrum* *Aspergillus amstelodami*	Sponges		Neoechinulin B 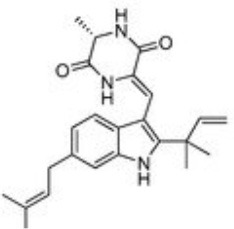	(17, 18)

Several therapeutic effects of neoechinulin A, B, and other derivatives have been reported in different studies including antioxidant, anti-inflammatory, antitumor effects, and neuroprotective activity (5, 16, 19–21).

**Figure 1:**
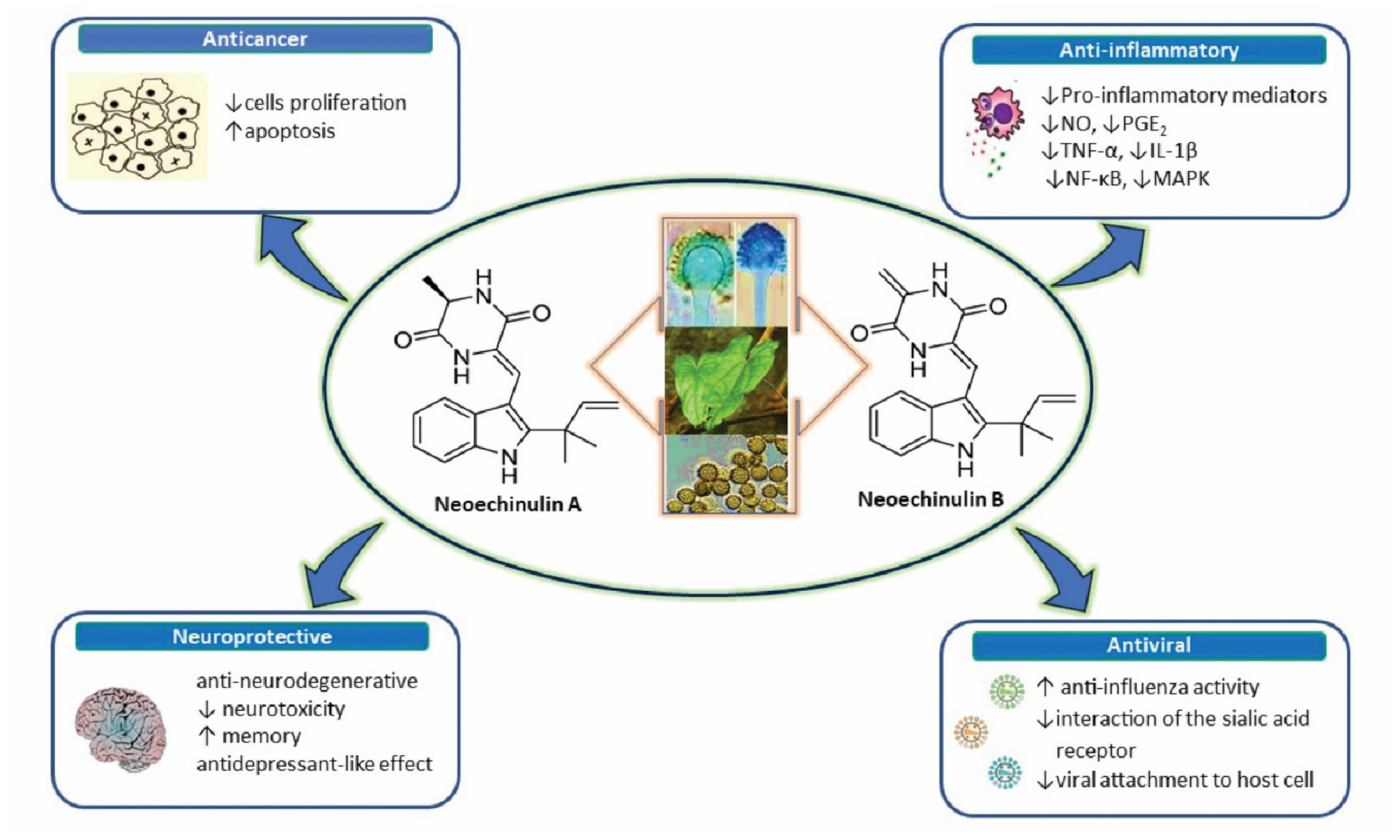
A diagram with the relevant pharmacological properties of the most important neoechinulins and their potential mechanism of actions. NO, nitric oxide; PGE, prostaglandin E2; TNF-α, tumour necrosis alfa; IL-1β, interleukin-1β; NF-Kβ, Nuclear factor-κβ; MAPK, mitogen-activated protein kinase.

In this paper, the aim is to review the therapeutic potential of neoechinulins together with their molecular mechanisms behind the biological activities, specifically focusing on preclinical pharmacological studies (Figure 1).

## Purposes and Review Methodology

### Search Strategy

A study was conducted on PubMed, ScienceDirect and Google scholar using various combinations of MESH terms and their synonyms: “neoechinulin,” “fungus,” “*in vivo*,” “*in vitro*,” “anti-inflammatory,” “anti-viral,” “anti- cancer ,” “neuroprotective.”

### Inclusion Criteria

- extensive works with isolated neoechinulins with the evaluation of pharmacological activities
- pre-clinical experimental pharmacological studies with determining doses and mechanisms of action
- studies and articles written only in the English language

### Exclusion Criteria

- studies that used in the experiment extracts from different parts of plants.
- studies in which neoechinulins have been associated with homoeopathic preparations or other nutritional supplements
- studies that included other substances, *in silico* studies, duplicates, abstracts

In this comprehensive review, we selected the best articles highlighting the possible molecular mechanisms of the pharmacological properties of neoechinulines. These data have been summarised in two figures and two tables.

## Pharmacological Activities of Neoechinulins

### Anti-inflammatory

Inflammation is an important defence mechanism of the body during acute inflammatory responses, maintaining tissue homeostasis (22, 23). However, sometimes uncontrolled acute inflammation might turn into chronic and lead to an array of medical conditions, including hepatitis, arthritis, and neurodegenerative ailments (24–26). In numerous inflammatory responses, macrophages reported to be activated by LPS 9 (lypopolisaccharides), produce pro-inflammatory mediators and cytokines *viz*. NO, interleukin-1β (IL-1β), PGE2 etc (27). These pro-inflammatory mediators and cytokines are induced by different enzymes like cyclooxygenase-2 (COX-2), iNOS (28, 29). Nuclear factor-kappa B (NF-κB) along with its inhibitory protein, IκB controls the transcription of pro-inflammatory mediators and cytokines such as NO (nitric oxide), iNOS (inducible isoform NO), COX-2 (ciclooxigenase 2), TNF-α (Tumour necrosis alfa), PGE2 (prostaglandin E2), and IL-1β (interleukin 1β) (30, 31) Besides, mitogen-activated protein kinases (MAPKs) are also known to induce production of cytokine and the expression of iNOS and COX-2 (32, 33).

Neoechinulin A exhibited anti-inflammatory activity (Table 1) on lipopolysaccharide (LPS)_-treated RAW264.7 macrophages which were implicated in the inhibition of NF-kB and p38 MAPK pathway (16). Besides, neoechinulin A induced reduction in the expression of iNOS catalyzes, COX-2 and MAPK pathway dose-dependently resulted in a dose-dependent decreased in the synthesis of nitric oxide (NO) and prostaglandin E2 (PGE2). This mechanism indicated that natural diketopiperazine-type indole alkaloids have anti-inflammatory properties, and their therapeutic potential can be used to treat inflammatory disorders (16). Moreover, neoechinulin A showed suppressive ability on Ab42-induced microglial activation and demonstrated capabilities to inhibit the production of neurotoxic inflammatory mediators (TNFα, IL-1β; IL-6; PGE2) in activated BV-2 cells through blocking the phosphorylation of MAPK. Therefore, results proved that neoechinulin A suppressed the production of reactive oxygen species (ROS) and reactive nitrogen species (RNS) remarkably and reduced the mRNA expressions and protein synthesis of various mediators of inflammation (34).

In another study, it has been reported that marine fungi produce natural metabolites that can reduce inflammation (35).

In another study, neoechinulins A and B were isolated from the marine fungus *Eurotium* sp. SF-5989, and their anti-inflammatory effects on lipopolysaccharide (LPS)-stimulated RAW264.7 macrophages were investigated. The results indicated that neoechinulin A inhibited the nitric oxide (NO) and prostaglandin E2 (PGE2) production as well as the inducible nitric oxide synthase (iNOS) and cyclooxygenase-2 (COX-2) expression, whereas neoechinulin B influenced the cell viability besides its similar suppressive effect on NO production at lower doses. Different mechanisms of action for neoechinulin A were also reported, indicating its potential as an anti-inflammatory agent (16).

### Anti-viral

Neoechinulin A, including emodin, emodin-8-O-β-D-glucoside, was isolated for the first time from the ethanolic root extract of *Aconitum carmichaelii* and was suggested for utilisation in discovering novel anti plant viral agents (14). Likewise, an aqueous extract of *Portulaca oleracea* showed the presence of neoechinulin D and possibilities for further utilisation for the treatment of diseases (15) (Table 1).

This information indicated that marine plants can produce neoechinulin and highlighted future gap area for further investigation.

Only ten diverse compounds *viz*., echinulin, neoechinulin A, neoechinulin D, dihydroauroglaucin, physcion, flavoglaucin, isodihydroauroglaucin, cinnalutein, asperflavin, and cyclo-L-Trp-L-Ala were isolated from *Eurotium. chevalieri*. Among these isolated compounds, neoechinulin D is reported with antiviral properties against herpes and inluenza viruses (17). Furthermore, neoechinulin B showed potency to inhibit the H1N1 virus-infected in MDCK cells (model mammalian cell line) and their ability to bind with influenza envelope hemagglutinin, breakdown of sialic acid receptor interaction and viral attachment to host cell. Therefore, neoechinulin B was cited as a potent inhibitor of the influenza virus (36) Similarly, in a study reporting neoechinulin B activity against HCV (hepatitis C virus) in the cell culture system, the compound was recorded as a novel suppressor of the LXR (liver X receptor). Neoechinulin B suppressed the induction of LXR-mediated transcription, disrupted double-membrane or multi membrane vesicles blocking the HCV replication and altered lipid metabolism. Thus, neoechinulin B can act as an anti-HCV agent without showing any type of cytotoxicity (18).

Neoechinulin B, a secondary metabolite of *Aspergillus amstelodami* was reported as a suppressor of the liver X receptor (LXR) in a cell culture system of the hepatitis C virus (HCV). The results of this study indicated that neoechinulin B suppresses LXR-mediated transcription, and interacts directly with LXRs (18).

In a recent study, a potent antiviral effect of neoechinulin B against H1N1 virus-infected in Madin-Darby canine kidney (MDCK) cells was also investigated. The authors reported that neoechinulin B was able to suppress influenza virus that included clinical isolates of amantadine- and oseltamivir-resistant strains. The mechanism was explained with the binding of neoechinulin B to influenza envelope hemagglutinin, disturbing its interaction with the sialic acid receptor and the virus attachment to host cells (37).

### Anti-cancer

**Figure 2:**
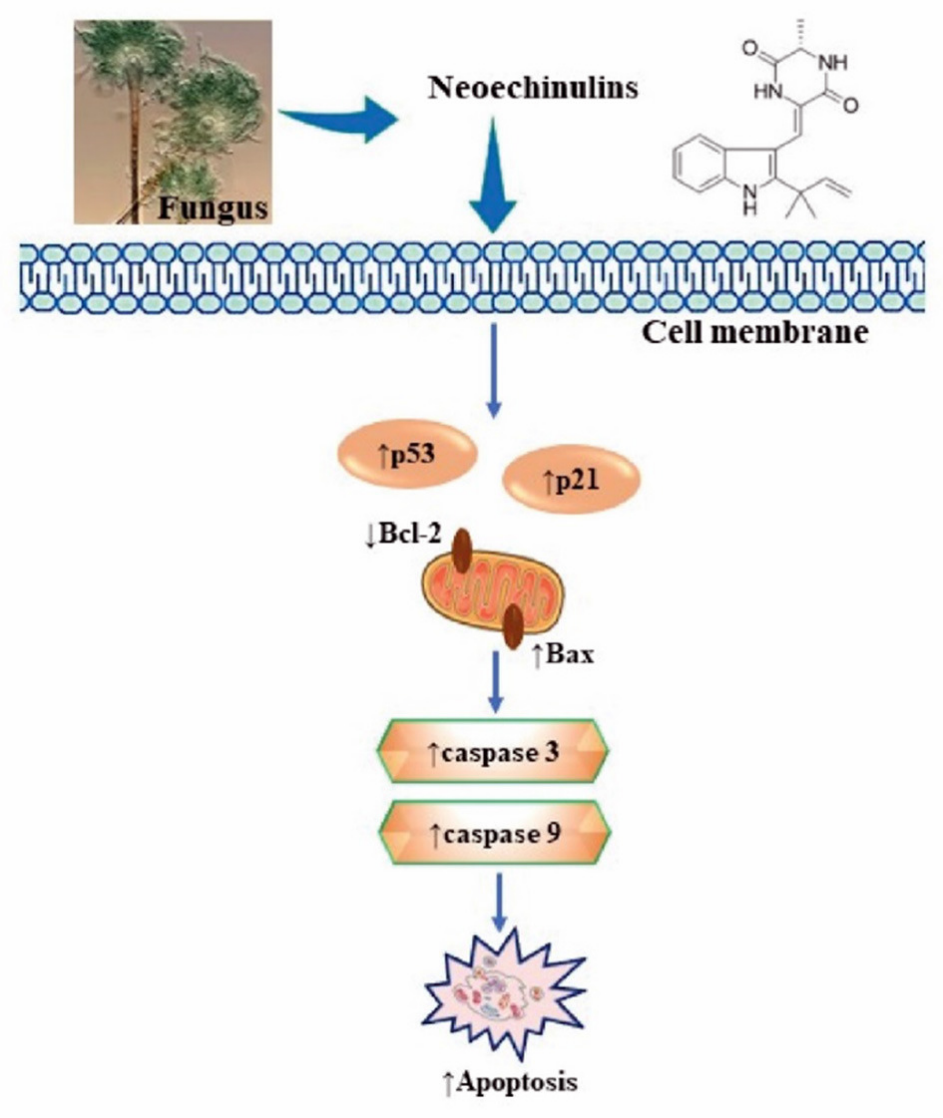
Summarised scheme of potential cytotoxic mechanisms of neochinulins to induce apoptosis in cancer cells. p53, p21, cellular tumour antigen; Bcl-2, B-cell lymphoma 2; Bax, Bcl-2-associated X Protein.

Neoechinulin A has the capabilities to act against different tumour cells (Figure 2) Most of the cancer cells block programmed cell death (apoptosis) through anti-apoptotic signalling pathways, thus disrupting the vital equilibrium between proliferation and death of the cells (38).

The expression of apoptosis-related proteins, in particular, anti-apoptotic proteins *viz*. Bcl-2, Bcl-xl, Mcl-1, and pro-apoptotic proteins *viz*. Bax, Bad, Bak is essential for apoptosis regulation (39). Study reported that neoechinulin A as an induced p53 tumour protein towards caspase-3 and thus enabled the HeLa cells to enter the apoptotic pathway. The results of this study showed that neoechinulins can down-regulate Bcl-2 protein expression and upregulate Bax protein expression, thus activating caspase-3 cascade and leading to the HeLa cells apoptosis. (40).

Anti-nitration, antioxidant activities and cytoprotection offered by neoechinulin A and its analogues against 3-morpholinosydnonimine (SIN-1)-induced phaeochromocytoma (PC12) cell death were measured to reveal the structure-activity relationships of these compounds (1) Z. The authors reported that the cytoprotective effect of an acyclic analogue was diminished while maintaining its antioxidant or anti-nitration properties. They have concluded that cytoprotective effect of neoechinulin A was provided by a mechanism not attributed to their antioxidant or anti-nitration properties (41).

### Neuroprotective

Neoechinulins displayed potent anti-neurodegenerative activities against neuro-inflammation, deposition of amyloid-β peptides in Alzheimer's disease, Parkinsonian-inducing neurotoxins and other cytotoxicity inducing neurotoxins (rotenone, MPP^+^, SIN-1, peroxynitrite) (Figure 2). Neoechinulin A showed cytoprotective properties in neuron-like PC12 cells subjected to a peroxynitrite-imposed oxidative injury (42). Besides, neoechinulin A protected PC12 cells from cytotoxic effects of 1-methyl-4-phenylpyridinium (MPP^+^), a potent neurotoxin provoking acute Parkinson's-like neurodegenerative symptoms in humans (43).

Several studies explored the biological potential of neoechinulin, in the treatment of neurodegenerative diseases (Table 2). Neurodegenerative diseases like Alzheimer's and Parkinson's diseases cause mortality and morbidity for millions of people across the globe (45). The predominant causative factors for these are mainly ageing and environmental (neurotoxins), apart from a small fraction of mutation related genes (46, 47). The main pathological changes related to these diseases are progressive and selective loss of dopaminergic neurons in the brain (48). This is mainly attributed to peroxynitrite, a potent oxidant generated in a biological system by the reaction of NO and superoxide anion (O^2−^) *in vivo* causing severe cell damage to neurons, by promoting the oxidation of various biomolecules (lipid, proteins and nucleic acids) (49–51) (Table 2).

**Table 2:** The most relevant pharmacological properties of neoechinulins, possible mechanisms, and signalling pathways.

**Source/Species**	**Compound**	**Experimental model**	**Possible molecular mechanisms**	**Effect**	**References**
Fungi/*Eurotium*sp. SF-5989	Neoechinulin A	LPS-stimulated RAW264.7 macrophages/*in vitro*	IC_50_ = 12.5–100 μM ↓pro-inflammatory mediators ↓cytokines, ↓NO, ↓PGE_2_, ↓TNF-α, ↓IL-1β, ↓ IκB-α, ↓NF-κB, ↓MAPK	Anti-inflammatory	(4, 16)
Fungi/*Eurotium rubrum*	Neoechinulin B	Infected MDCK cells with Influenza virus/*in vitro*	IC_50_ = 27.4 μM Binding with influenza envelope hemagglutinin → ↑ anti-influenza activity ↓interaction of the sialic acid receptor ↓viral attachment to host cell	Antiviral	(37)
Fungi/*Eurotiumchevalieri* MUT 2316	Neoechinulin D	MDCK cells/*in vitro*	↓viral replication of IAV or HSV-1	Antiviral	(17)
Fungi	Neoechinulin A	PC12 cells/*in vitro*	IC_50_ = 100 mM Neuroprotection against Parkinson's disease-inducing rotenone neurotoxin ↑ATP consumption in cells	Cytoprotective of neuronal cells	(38)
Fungi/*Microsporum* sp.	Neoechinulin A	HeLa cells/*in vitro*	IC_50_ = 1.25–10 μM ↓proliferation, ↑apoptosis ↓anti-apoptotic protein Bcl-2 ↑pro-apoptotic proteins Bax ↑p53, ↑p21, ↑caspase3, ↑ caspase 9	Anticancer	(40)
Fungi/*Eurotium rubrum*	Neoechinulin A	PC12 cells/*in vitro* Cytotoxicity induced by SIN-1	IC_50_ = 40 μM ↑affinity for proteins chromogranin B and glutaredoxin 3 ↓neuro-cytotoxicity	Neuroprotective	(44)
Fungi	Neoechinulin A	PC12 cells/*in vitro*	IC_50_ = 200 μM ↓SIN-1- induced activation of caspase-3–like proteases, ↑NADH-dehydrogenase, ↓ROS, ↓neuronal cell death	Neuroprotective	(42)

*↓decreased, ↑increased, → results*.

Marine-derived natural products such as neoechinulins produce a variety of pharmacological effects for downstream amelioration of neurotoxins-induced cytotoxicity. Its pharmacological properties against various neurotoxins are generally attributed to the C8/C9 double bond, offering a conjugated system across the indole moiety to the diketopiperazine ring. Additionally, the antioxidant efficacy or electrophilic nature of the C-8 carbon may contribute to the cytoprotective ability of the alkaloid (19). However, diketopiperazine moiety is reported as a requisite for anti-nitration activity (38).

Neoechinulin A protects neuronal PC12 cells (cell line from rat adrenal medulla) against cell death imposed by peroxynitrite derived from SIN 1 [3-(4-morpholinyl) sydnonimine hydrochloride] (42). Neoechinulin A exhibited a strong affinity for specific binding to diverse proteins, namely chromogranin B and glutaredoxin 3. This high affinity of neoechinulin A might be responsible for the protection of PC12 cells from cytotoxicity by inducing S–morpholinosydnonimine (SIN-1). Neoechinulin, apart from its apparent antioxidant and anti-nitration activities, imparts cytoprotection to cells with neurotrophic factor-like and anti-apoptotic properties, probably via potentiation of NAD(P)H-producing ability of the cell (44). However, the NAD(P)H-generating dehydrogenase(s) involved in the process remain elusive (41).

Neoechinulin A treatment can be useful for the protection of PC12 cells against MPP^++^ neurotoxin cytotoxicity derived from prodrug 1-methyl-4-phenyl-1,2,3,6 tetrahydropyridine (MPTP) (52). The MPP^++^ which assembles in mitochondria and binds to electron transport chain's complex I, thus reducing ATP biosynthesis, leads to cell death; is ameliorated by an increase in the ability of cells to produce NADH (43). Furthermore, the increase in NADH or lowering of cellular ATP levels by Neoechinulin A is also found to be effective against Parkinsonian-inducing neurotoxins such as rotenone (a pesticide), targeting mitochondrial complex I in animal models such as rat pheochromocytoma cell line(38), thereby delaying the progression of neurodegenerative diseases. In another study, synthetic (–) and (+) neoechinulin A, displayed cytoprotection by preventing SIN-1-induced cytotoxicity in nerve growth factor (NGF)-differentiated PC12 cells. The structure-activity relationships explained that the C8/C9 double bond played a crucial role attributed to its anti-cytotoxic potential (21).

Neoechinulin A was also investigated for its efficacy on cognitive damage in mice treated with lipopolysaccharide (LPS) mice and its antidepressive properties in were also recorded. Neoechinulin A enhanced cognitive ability in LPS-insulted mice besides offering antidepressant-like properties (53) (Table 2).

## Overall Conclusion and Future Perspectives

Neoechinulins and their analogues, derived from marine fungi, with proven pharmacological benefits for human health, can be prolific therapeutic agents for many existing diseases. Due to these positive pharmacological effects, neoquinolines derived from marine fungi can be considered in the future as potential functional and integrative nutrition, as part of functional and integrative medicine, with a major role in preventing, reducing causes, manifestations of diseases through a diet appropriate to each patient. In the future, neoquinolines may be introduced into functional foods or dietary compounds (nutraceuticals) that benefit human health by preventing or treating diseases, or by correcting metabolic disorders, or by preventing the progression or recurrence of a pathological situation.

The therapeutic applications of neoechinulins and their natural and synthetic analogues and derivatives are needed to be explored by more animal models via analysing their structure-activity relationships and elucidating their underlying mechanisms of action specific to various biosynthesis and signalling pathways and possible cross-talks through high throughout techniques. Although primarily obtained from fungal sources, such compounds have also been discovered in higher plants. Numerous researches have demonstrated their potential as anti-inflammatory, anti-viral, anti-cancerous and anti-Parkinson's properties. Besides, they exhibited potent anti-neurodegenerative efficacy against neuroinflammation and neurotoxicity. Structure-activity relationships of neoechinulins and their analogues with their reported bioactivities are in their nascent stage and are needed to be elucidated. The reported antioxidant or electrophilic property of the C-8 carbon, both of which are contributed by the double bond in C-8/C-9, is probably attributed to the cytoprotective nature of this alkaloid. Besides, diketopiperazine moiety was reported as a requisite for anti-nitration activity. However, this potential, along with ever-expanding microbial resistance to conventional drugs and quest for the search for novel natural compounds of pharmacological importance, is to be complemented by an investigation of the specific biosynthesis pathway, signalling pathway through high throughput techniques.

## Author Contributions

JS-R, ADe, EC, AB, PD, PS, and ADo contributed significantly to analysis and manuscript preparation. ADe, JS-R, AA-H, and DC assistance to the revision of the manuscript. ADe, AA-H, JS-R, and ADo supported valuable discussion. JS-R, ADo, and DC revised the whole manuscript. All authors collated documents, wrote the manuscript, read, and approved the final manuscript.

## Conflict of Interest

The authors declare that the research was conducted in the absence of any commercial or financial relationships that could be construed as a potential conflict of interest.

## Abbreviations

COX-2: cyclooxygenase-2
HCV: hepatitis C virus
5-HT: 5-hydroxytryptamine
iNOS: inducible nitric oxide synthase
IκB: inhibitor of nuclear factor-kappa B
IL-1β: interleukin-1β
i.c.v.: intracerebroventricular
LPS: lipopolysaccharide
LXR: liver X receptor
MDCK: Madin-Darby canine kidney
MAPKs: mitogen-activated protein kinases
MPTP: 1-methyl-4-phenyl-1,2,3,6 tetrahydropyridine
MPP^+^: 1-methyl-4-phenylpyridinium
NGF: nerve growth factor
NO: nitric oxide
NF-κB: nuclear factor-kappa B
O^2−^: superoxide anion
PC12: phaeochromocytoma
PGE2: prostaglandin E2
RNS: reactive nitrogen species
ROS: reactive oxygen species
SIN-1: 3-morpholinosydnonimine.

## References

[1] Yamamoto Y Arai K. Alkaloidal substances from aspergillus species. In: Arnold B editor. The Alkaloids: Chemistry And Pharmacology. Maryland: Elsevier. (1986). 10.1016/S0099-9598(08)60249-7

[2] Dossena A Marchelli R Pochini A. New metabolites of aspergillus amstelodami related to the biogenesis of neoechinulin. Chem Commun. (1974) 771–2. 10.1039/c39740000771

[3] Marchelli R Dossena A Pochini A Dradi E. The structures of five new didehydropeptides related to neoechinulin, isolated from aspergillus amstelodami. J Chem Soc Perkin. (1977) 1:713–7. 10.1039/p19770000713 558214

[4] Li Y Li X Kang JS Choi HD Son BW. New radical scavenging and ultraviolet-a protecting prenylated dioxopiperazine alkaloid related to isoechinulin a from a marine isolate of the fungus aspergillus. J Antibiot. (2004) 57:337–40. 10.7164/antibiotics.57.337 15303494

[5] Yagi R Doi M. Isolation of an antioxidative substance produced by aspergillus repens. Biosci Biotechnol Biochem. (1999) 63:932–3. 10.1271/bbb.63.932 27385574

[6] Wang XN Tan RX Liu JK. Xylactam, a new nitrogen-containing compound from the fruiting bodies of ascomycete xylaria euglossa. J Antibiot. (2005) 58:268–70. 10.1038/ja.2005.31 15981413

[7] Wang S Li XM Teuscher F Li DL Diesel A Ebel R. Chaetopyranin, a benzaldehyde derivative, and other related metabolites from chaetomium globosum, an endophytic fungus derived from the marine red alga polysiphonia urceolata. J Nat Prod. (2006) 69:1622–5. 10.1021/np060248n 17125234

[8] Du FY Li XM Li CS Shang Z Wang BG. Cristatumins A-D, new indole alkaloids from the marine-derived endophytic fungus eurotium cristatum En-220. Bioorg Med Chem Lett. (2012) 22:4650–3. 10.1016/j.bmcl.2012.05.088 22727636

[9] Slack GJ Puniani E Frisvad JC Samson RA Miller JD. Secondary metabolites from eurotium species, aspergillus calidoustus and A. Insuetus common in canadian homes with a review of their chemistry and biological activities. Mycol Res. (2009) 113:480–90. 10.1016/j.mycres.2008.12.002 19422073

[10] Yan X Zhou Y Tang J Ji M Lou H Fan P. Diketopiperazine indole alkaloids from hemp seed. Acs Omega. (2016) 18:77–82. 10.1016/j.phytol.2016.09.001

[11] Sun YT Wang AL Li DH Li ZL Liu XQ Hua HM. Nitrogeous chemical constituents from tinosporae radix. Chin Tradit Herb Drugs. (2015) 46:1287–91.

[12] Wu Q Hua HM Li ZL. Isolation and identification of the chemical constituents of opuntia dillenii haw. Chin J Med Chem. (2013) 2.

[13] Yang SJ Liu MC Liang N Xiang H-M Yang S. Chemical constituents of cyrtomium fortumei smith. Nat Prod Res. (2013) 27:2066–8. 10.1080/14786419.2013.824442 23962117

[14] Zhu YY Yu G Wang YY Xu JH Xu FZ Fu H. Antiviral activity and molecular docking of active constituents from the root of aconitum carmichaelii. Nat Prod Res. (2019) 55:189–93. 10.1007/s10600-019-02651-5

[15] Zhao C Zhang C He F Zhang W Leng A Ying X. Two new alkaloids from portulaca oleracea L. And their bioactivities. Fitoterapia. (2019) 136:104166. 10.1016/j.fitote.2019.05.005 31075485

[16] Kim KS Cui X Lee DS Sohn JH Yim JH Kim YC. Anti-Inflammatory effect of neoechinulin a from the marine fungus *Eurotium* Sp. Sf-5989 through the suppression of NF-κB and P38 MAPK pathways in lipopolysaccharide-stimulated RAW264 7 macrophages. Molecules. (2013) 18:13245–59. 10.3390/molecules181113245 24165583 PMC6270177

[17] Bovio E Garzoli L Poli A Luganini A Villa P Musumeci R. Marine fungi from the sponge grantia compressa: biodiversity, chemodiversity, and biotechnological potential. Mar Drugs. (2019) 17:220. 10.3390/md17040220 30978942 PMC6520677

[18] Nakajima S Watashi K Ohashi H Kamisuki S Izaguirre-Carbonell J Kwon AT-J. Fungus-derived neoechinulin b as a novel antagonist of liver X receptor, identified by chemical genetics using a hepatitis C virus cell culture system. J Virol. (2016) 90:9058–74. 10.1128/JVI.00856-16 27489280 PMC5044839

[19] Kuramochi K Ohnishi K Fujieda S Nakajima M Saitoh Y Watanabe N. Synthesis and biological activities of neoechinulin a derivatives: new aspects of structure–activity relationships for neoechinulin A. Chem Pharm Bull. (2008) 56:1738–43. 10.1248/cpb.56.1738 19043251

[20] Pettit GR Hogan F Xu J-P Tan R Nogawa T Cichacz Z. Antineoplastic agents. 536. New sources of naturally occurring cancer cell growth inhibitors from marine organisms. Terrestrial plants, and microorganisms. J Nat Prod. (2008) 71:438–44. 10.1021/np700738k 18327911

[21] Aoki T Ohnishi K Kimoto M Fujieda S Kuramochi K Takeuchi T. Synthesis and neuroprotective action of optically pure neoechinulin A and its analogs. Pharmaceuticals. (2010) 3:1063–9. 10.3390/ph3041063 27713287 PMC4034020

[22] Sharifi-Rad J Rodrigues CF Sharopov F Docea AO Karaca AC Sharifi-Rad M. Diet, lifestyle and cardiovascular diseases: linking pathophysiology to cardioprotective effects of natural bioactive compounds. Int J Environ Res Public Health. (2020) 17:2326. 10.3390/ijerph17072326 32235611 PMC7177934

[23] Sharifi-Rad M Kumar NVA Zucca P Varoni EM Dini L Panzarini E. Lifestyle, oxidative stress, and antioxidants: back and forth in the pathophysiology of chronic diseases. Front Physiol. (2020) 11:694. 10.3389/fphys.2020.00694 32714204 PMC7347016

[24] Salehi B Capanoglu E Adrar N Catalkaya G Shaheen S Jaffer M. Cucurbits plants: a key emphasis to its pharmacological potential. Molecules. (2019) 24:1854. 10.3390/molecules24101854 31091784 PMC6572650

[25] Padureanu R Albu CV Mititelu RR Bacanoiu MV Docea AO Calina D. Oxidative stress and inflammation interdependence in multiple sclerosis. J Clin Med. (2019) 8:1815. 10.3390/jcm8111815 31683787 PMC6912446

[26] Sharifi-Rad M Lankatillake C Dias DA Docea AO Mahomoodally MF Lobine D. Impact of natural compounds on neurodegenerative disorders: from preclinical to pharmacotherapeutics. J Clin Med. (2020) 9:1061. 10.3390/jcm9041061 32276438 PMC7231062

[27] Salehi B Rescigno A Dettori T Calina D Docea AO Singh L. Avocado-Soybean unsaponifiables: a panoply of potentialities to be exploited. Biomolecules. (2020) 10:130. 10.3390/biom10010130 31940989 PMC7023362

[28] Salehi B Shetty MS Kumar NVA Zivkovic J Calina D Docea AO. Veronica plants-drifting from farm to traditional healing, food application, and phytopharmacology. Molecules. (2019) 24:2454. 10.3390/molecules24132454 31277407 PMC6651156

[29] Salehi B Sestito S Rapposelli S Peron G Calina D Sharifi-Rad M. epibatidine: a promising natural alkaloid in health. Biomolecules. (2019) 9:6. 10.3390/biom9010006 30583611 PMC6359223

[30] Mititelu RR Padureanu R Bacanoiu M Padureanu V Docea AO Calina D. Inflammatory and oxidative stress markers-mirror tools in rheumatoid arthritis. Biomedicines. (2020) 8:125. 10.3390/biomedicines8050125 32429264 PMC7277871

[31] Salehi B Sharifi-Rad J Cappellini F Reiner A Zorzan D Imran M. The therapeutic potential of anthocyanins: current approaches based on their molecular mechanism of action. Front Pharmacol. (2020) 11:1300. 10.3389/fphar.2020.01300 32982731 PMC7479177

[32] Sharifi-Rad J Kamiloglu S Yeskaliyeva B Beyatli A Alfred MA Salehi B. Pharmacological activities of psoralidin: a comprehensive review of the molecular mechanisms of action. Front Pharmacol. (2020) 11:571459. 10.3389/fphar.2020.571459 33192514 PMC7643726

[33] Salehi B Mishra AP Nigam M Karazhan N Shukla I Kieltyka-Dadasiewicz A. Ficusplants: state of the art from a phytochemical, pharmacological, and toxicological perspective. Phytother Res. (2021) 35:1187–217. 10.1002/ptr.6884 33025667

[34] Dewapriya P Li YX Himaya S Pangestuti R Kim SK. Neoechinulin A suppresses amyloid-β oligomer-induced microglia activation and thereby protects Pc-12 cells from inflammation-mediated toxicity. Neurotoxicology. (2013) 35:30–40. 10.1016/j.neuro.2012.12.004 23261590

[35] Xu J Yi M Ding L He S. A review of anti-inflammatory compounds from marine fungi, 2000–2018. Mar Drugs. (2019) 17:636. 10.3390/md17110636 31717541 PMC6891400

[36] Abdelmohsen UR Balasubramanian S Oelschlaeger TA Grkovic T Pham NB Quinn RJ. Potential of marine natural products against drug-resistant fungal, viral, and parasitic infections. Lancet Infect Dis. (2017) 17:E30–41. 10.1016/S1473-3099(16)30323-1 27979695

[37] Chen X Si L Liu D Proksch P Zhang L Zhou D. Neoechinulin B and its analogues as potential entry inhibitors of influenza viruses, targeting viral hemagglutinin. Eur J Med Chem. (2015) 93:182–95. 10.1016/j.ejmech.2015.02.006 25681711

[38] Akashi S Kimura T Takeuchi T Kuramochi K Kobayashi S Sugawara F. Neoechinulin A impedes the progression of rotenone-induced cytotoxicity in Pc12 cells. Biol Pharm Bull. (2011) 34:243–8. 10.1248/bpb.34.243 21415535

[39] Porter AG Jänicke RU. Emerging roles of caspase-3 in apoptosis. Cell Death Differ. (1999) 6:99–104. 10.1038/sj.cdd.4400476 10200555

[40] Wijesekara I Li YX Vo TS Van Ta Q Ngo DH Kim SK. Induction of apoptosis in human cervical carcinoma hela cells by neoechinulin a from marine-derived fungus microsporum Sp. Food Chem Toxicol. (2013) 48:68–72. 10.1016/j.procbio.2012.11.012

[41] Kimoto K Aoki T Shibata Y Kamisuki S Sugawara F Kuramochi K. Structure-activity relationships of neoechinulin A analogues with cytoprotection against peroxynitrite-induced Pc12 cell death. J Antibiot. (2007) 60:614–21. 10.1038/ja.2007.79 17965477

[42] Maruyama K Ohuchi T Yoshida K Shibata Y Sugawara F Arai T. protective properties of neoechinulin a against sin-1–induced neuronal cell death. J Biochem. (2004) 136:81–7. 10.1093/jb/mvh103 15269243

[43] Kajimura Y Aoki T Kuramochi K Kobayashi S Sugawara F Watanabe N. Neoechinulin A protects Pc12 cells against MPP+-induced cytotoxicity. J Antibiot (Tokyo). (2008) 61:330–3. 10.1038/ja.2008.48 18654001

[44] Kamisuki S Himeno N Tsurukawa Y Kusayanagi T Takeno M Kamakura T. Identification of proteins that bind to the neuroprotective agent neoechinulin A. Biosci Biotechnol Biochem. (2018) 82:442–8. 10.1080/09168451.2018.1433018 29447077

[45] Calina D Buga AM Mitroi M Buha A Caruntu C Scheau C. The treatment of cognitive, behavioural and motor impairments from brain injury and neurodegenerative diseases through cannabinoid system modulation-evidence from in vivo studies. J Clin Med. (2020) 9:28. 10.3390/jcm9082395 32726998 PMC7464236

[46] Dawson TM Dawson VL. Molecular pathways of neurodegeneration in Parkinson's disease. Science. (2003) 302:819–22. 10.1126/science.1087753 14593166

[47] Gorman AM. Neuronal cell death in neurodegenerative diseases: recurring themes around protein handling. J Cell Mol Med. (2008) 12:2263–80. 10.1111/j.1582-4934.2008.00402.x 18624755 PMC4514105

[48] Huang C Zhang Z Cui W. Marine-Derived natural compounds for the treatment of Parkinson's disease. Mar Drugs. (2019) 17:221. 10.3390/md17040221 30978965 PMC6520879

[49] Beckman JS Ischiropoulos H Zhu L Van Der Woerd M Smith C Chen J. Kinetics of superoxide dismutase- and iron-catalyzed nitration of phenolics by peroxynitrite. Arch Biochem Biophys. (1992) 298:438–45. 10.1016/0003-9861(92)90432-V 1416975

[50] Smith MA Richey Harris PL Sayre LM Beckman JS Perry G. Widespread peroxynitrite-mediated damage in Alzheimer's disease. J Neurosci. (1997) 17:2653–7. 10.1523/JNEUROSCI.17-08-02653.1997 9092586 PMC6573097

[51] Good PF Hsu A Werner P Perl DP Olanow CW. Protein nitration in Parkinson's disease. J Neuropathol Exp Neurol. (1998) 57:338–42. 10.1097/00005072-199804000-00006 9600227

[52] Langston JW Ballard P Tetrud JW Irwin I. Chronic parkinsonism in humans due to a product of meperidine-analog synthesis. Science. (1983) 219:979–80. 10.1126/science.6823561 6823561

[53] Sasaki-Hamada S Hoshi M Niwa Y Ueda Y Kokaji A Kamisuki S. Neoechinulin A induced memory improvements and antidepressant-like effects in mice. Prog Neuropsychopharmacol Biol Psychiatry. (2016) 71:155–61. 10.1016/j.pnpbp.2016.08.002 27495355

